# Impact of time from biopsy to surgery on complications, functional and oncologic outcomes following radical prostatectomy

**DOI:** 10.1590/S1677-5538.IBJU.2018.0196

**Published:** 2019-07-27

**Authors:** Mary E. Westerman, Vidit Sharma, George C. Bailey, Stephen A. Boorjian, Igor Frank, Matthew T. Gettman, R. Houston Thompson, Matthew K. Tollefson, Robert Jeffrey Karnes

**Affiliations:** 1 Department of Urology, Mayo Clinic, Rochester, Minnesota, USA

**Keywords:** Surgical Procedures, Operative, Prostatectomy, Therapeutics

## Abstract

**Introduction:**

To determine the impact of time from biopsy to surgery on outcomes following radical prostatectomy (RP) as the optimal interval between prostate biopsy and RP is unknown.

**Material and methods:**

We identified 7, 350 men who underwent RP at our institution between 1994 and 2012 and had a prostate biopsy within one year of surgery. Patients were grouped into five time intervals for analysis: ≤ 3 weeks, 4-6 weeks, 7-12 weeks, 12-26 weeks, and > 26 weeks. Oncologic outcomes were stratified by NCCN disease risk for comparison. The associations of time interval with clinicopathologic features and survival were evaluated using multivariate logistic and Cox regression analyses.

**Results:**

Median time from biopsy to surgery was 61 days (IQR 37, 84). Median follow-up after RP was 7.1 years (IQR 4.2, 11.7) while the overall perioperative complication rate was 19.7% (1,448/7,350). Adjusting for pre-operative variables, men waiting 12-26 weeks until RP had the highest likelihood of nerve sparing (OR: 1.45, p = 0.02) while those in the 4-6 week group had higher overall complications (OR: 1.33, p = 0.01). High risk men waiting more than 6 months had higher rates of biochemical recurrence (HR: 3.38, p = 0.05). Limitations include the retrospective design.

**Conclusions:**

Surgery in the 4-6 week time period after biopsy is associated with higher complications. There appears to be increased biochemical recurrence rates in delaying RP after biopsy, for men with both low and high risk disease.

## INTRODUCTION

In 2018, it is estimated that 164.690 men in the United States will be diagnosed with prostate cancer (PCa) and 29.430 will die of the disease (1). Localized prostate cancer is diagnosed by trans-rectal ultrasound guided biopsy (2). Following diagnosis of local disease, approximately 50% of men elect radical prostatectomy (RP) for definitive treatment (3). Traditionally, urologists have recommended an interval of at least 4 to 8 weeks between prostate biopsy and RP in order to allow inflammation to abate.

However, the impact of time interval between biopsy and RP has not been well established (4–9). While some observations have suggested early RP is associated with more overall complications and a greater risk of blood transfusion (8), others have found disparate results (5, 7, 9). Herein, we assess the impact of time from biopsy to surgery on complications, functional, and oncologic outcomes following radical prostatectomy.

## MATERIALS AND METHODS

Following Institutional Review Board approval, we reviewed our Prostatectomy Registry and identified 15, 913 men who underwent RP between 1994 to 2012 at Mayo Clinic. Men with clinical T4 or metastatic disease, those who received neoadjuvant therapy, those with no biopsy date available and those who waited more than a year between biopsy and RP were excluded. The analysis was limited to the top 5 surgeons by volume to account for the fact that higher volume surgeons often have longer wait times. Each completed at least 900 cases during the study period. This left a cohort of 7.350 men with cT1 - 3N0 prostate cancer who underwent prostate biopsy within one year of RP and were included in the study.

The date of prostate biopsy performed closest to surgery was abstracted. As a tertiary referral center, many patients have their biopsy done elsewhere. However because we routinely have the pathology re - reviewed at our center, we were able to obtain the date of the original biopsy. Additional clinicopathologic features recorded included age at surgery, year of surgery, body mass index (BMI), prostate volume, preoperative PSA, number of previous prostate biopsies, clinical tumor stage according to the American Joint Committee on Cancer 2010 staging (10), pathologic TNM stage, Gleason score at RP, margin status, tumor volume, and receipt of adjuvant as well as salvage radiotherapy and androgen deprivation therapy.

Operative features including surgical approach, units of blood transfused, intraoperative and postoperative complications and functional outcomes at 1 year were obtained. Continence was defined as 0 pads per day use. Erectile function is defined as the ability to achieve an erection adequate for intercourse with or without the use of PDE - 5 inhibitors. For these evaluations, men incontinent and / or impotent prior to surgery were excluded.

Postoperative follow-up, including physical examination and serum PSA measurement, was not standardized given the retrospective nature of the cohort, but was generally performed quarterly for the initial 2 years, semiannually for the next 2 years, and annually thereafter. BCR was defined as a single postoperative PSA of 0.4 ng /mL or greater (11, 12). For men followed elsewhere, the Prostatectomy Registry monitors outcomes annually by correspondence.

Patients were grouped into 5 time periods for analysis: ≤ 3 weeks (n = 971, 13.2%), 4 - 6 weeks (n = 1179, 16.0%), 7 - 12 weeks (n = 3375, 45.9%), 13 - 26 weeks (n = 1615, 22.0%), and > 26 weeks (n = 210, 2.9%). Oncologic outcomes were stratified by NCCN criteria (low, intermediate, or high) (13). Continuous features were summarized with means and standard deviation, and compared using analysis of variance (ANOVA). Categorical features were summarized with frequency counts and percentages. Significance of differences across groups was assessed with the χ2 test for categorical variables. Multivariable logistic regression was performed to assess the association of time from biopsy to RP on complications, functional outcomes, and oncologic outcomes. Propensity score matching was not preferred in our analysis due to our event to confounder ratio > 8: 1 for all outcomes of interest (14). Results are summarized with odds ratios (OR) or hazard rations (HR) and 95% confidence intervals (CI). Statistical analysis was performed using SPSS software package (V.22 IBM: Aramonk, NY). All tests were two - sided, with p < 0.05 considered statistically significant.

## RESULTS

7.350 men with cT1 - 3N0 prostate cancer underwent prostate biopsy a median of 61 days [IQR 37, 84]) prior to RP. Clinicopathologic features, stratified by time from biopsy to RP, are presented in Table-1. As can be seen, men who underwent RP within 3 weeks of biopsy were older (mean age 63.3 vs. 61.0 years; p < 0.001), with a higher pre-operative PSA (8.6 ng / dL vs. 6.1 ng / dL; p < 0.001) and clinically higher risk disease (15.3% vs. 3.8% with NCCN high risk disease; p < 0.001) than those who waited 13 - 26 weeks.

**Table 1:** Clinicopathologic Characteristics stratified by time from biopsy to surgery.

		Overall Cohort† (N=7350)	≤ 3 weeks (N=971 (%))	4-6 weeks (N=1179) (%)	7-12 weeks (N=3375) (%)	12-26 weeks (N=1615) (%)	>26 weeks (N=210) (%)	p-value
**Mean age at surgery (SD)**		61.5±7.1	63.3±7.3	62.7±7.1	60.8±7.1	61.0±6.8	62.3±6.3	<0.001*
**Mean BMI at surgery (kg/cm^2^) (SD)**		28.1±4.0	28.3±3.9	28.6±4.0	27.9±3.9	28.3±4.1	28.3±4.0	<0.001*
**Mean preoperative PSA (ng/mL) (SD)**		7.1±7.3	8.6±11.7	8.3±9.0	6.7±5.9	6.1±4.6	6.5±5.0	<0.001*
**Clinical stage (%)**								<0.001
	cT1	4380 (60.2)	428 (44.5)	564 (48.4)	2128 (63.6)	1107 (69.5)	153 (74.3)	
	cT2	2778 (38.2)	513 (53.3)	558 (47.9)	1179 (35.2)	477 (30.0)	51 (24.8)	
	cT3	115 (1.6)	21 (2.2)	44 (3.8)	40 (1.2)	8 (0.5)	2 (1.0)	
**Biopsy Gleason score (%)**								<0.001
	≤6	5035 (69.0)	654 (68.5)	721 (61.9)	2276 (67.6)	1205 (75.3)	179 (85.2)	
	7	1882 (25.8)	212 (22.2)	344 (29.5)	936 (27.8)	362 (22.6)	28 (13.3)	
	8-10	380 (5.2)	89 (9.3)	100 (8.6)	154 (4.6)	34 (2.1)	3 (1.4)	
**TRUS volume (SD)**		37.9±19.9	38.6±20.7	37.5±19.9	37.3±19.7	38.6±19.7	41.2±19.4	0.01*
**NCCN Risk Group (%)**								<0.001
	Low	3936 (53.6)	418 (43.0)	494 (41.9)	1838 (54.5)	1040 (64.4)	146 (69.5)	
	Intermediate	2774 (37.7)	404 (41.6)	512 (43.4)	1288 (38.2)	514 (31.8)	56 (26.7)	
	High	640 (8.7)	149 (15.3)	173 (14.7)	249 (7.4)	61 (3.8)	8 (3.8)	
**Pathological Gleason score (%)**								<0.001
	≤ 6	4249 (58.0)	606 (62.5)	630 (53.5)	1914 (56.8)	971(60.5)	128 (61.2)	
	7	2632 (35.9)	268 (27.6)	428 (36.4)	1277 (37.9)	584 (36.4)	75 (35.9)	
	8 – 10	449 (6.1)	96 (9.9)	119 (10.1)	177 (5.3)	51 (3.2)	6 (2.9)	
**Surgeon**								<0.001
	**1**	2627 (35.7)	193 (19.9)	202 (17.1)	1585 (47.0)	575 (35.6)	72 (34.3)	
	**2**	1109 (15.1)	329 (33.9)	392 (33.2)	305 (9.0)	69 (4.3)	14 (6.7)	
	**3**	1354 (18.4)	414 (42.6)	433 (36.7)	358 (10.6)	118 (7.3)	31 (14.8)	
	**4**	1309 (17.8)	28 (2.9)	92 (7.8)	594 (17.6)	539 (33.4)	56 (26.7)	
	**5**	951 (12.9)	7 (0.7)	60 (5.1)	533 (15.8)	314 (19.4)	37 (17.6)	
**Robotic (%)**		2055 (28.0)	21 (2.2)	107 (9.1)	1014 (30.0)	824 (51.0)	89 (42.4)	<0.001
**Mean Blood loss (cc) (SD**)		828.8±1867.8	965.8±2019.6	843.6±1770.2	845.4±1918.8	700.0±1708.2	837.1±1974.3	0.01*
**pN1 (SD)**		184 (2.5)	38 (3.9)	55 (4.7)	72 (2.1)	16 (1.0)	3 (1.4)	<0.001
**Mean time to death (years) (SD)**		8.2±4.8	12.0±4.7	10.0±4.9	7.6±4.4	5.9±3.7	6.4±4.7	<0.001

* Means compared using ANOVA

**Figure 1:**
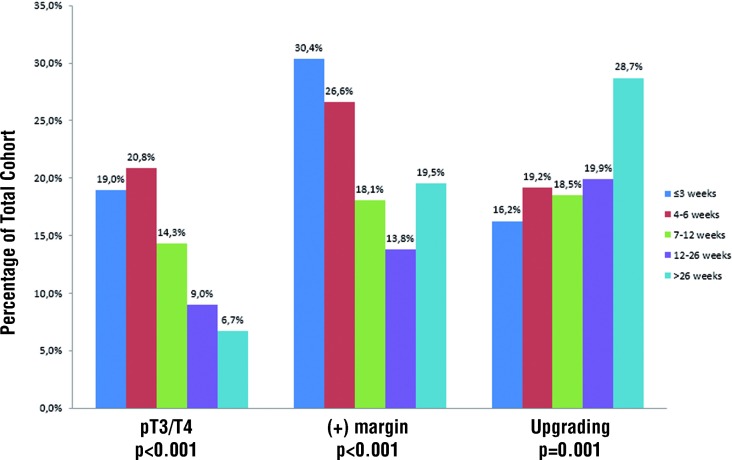
Pathologic outcomes stratified by Time from Biopsy to Radical Prostatectomy.

At RP, patients who had early intervention were more likely to have adverse pathologic features and were less likely to receive a nerve sparing (partial or complete) operation. Specifically, those who were biopsied within 3 weeks of surgery were more likely to have Gleason score 8 - 10 (9.9% vs. 3.2%; p < 0.001), pT3 / T4 disease (19.0% vs. 9.0%; p < 0.001), and a positive margin (30.4% vs. 13.8%; p < 0.001) compared to men waiting 13 - 26 weeks (Figure-1). Only 65.9% of those undergoing RP within 3 weeks received partial or complete nerve sparing versus 92.5% of men waiting at least 12 weeks (p < 0.001).

**Figure 2:**
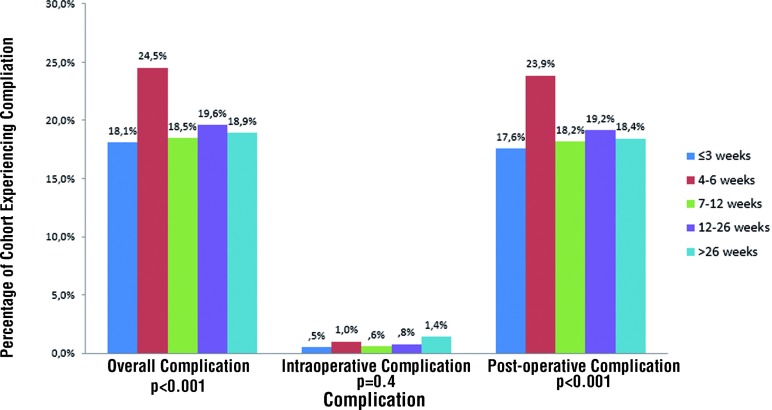
Complications stratified by time from Biopsy to Radical Prostatectomy.

Perioperative complications stratified by time since biopsy are shown in Figure-2. The overall complication rate was 19.7% with a 0.7% intraoperative complication rate. Complications included both early (≤ 30 days post - RP) and late (> 30 days post - RP) events such as lymphocele, urine leak, urinary tract infection, bladder neck contracture, deep vein thrombosis / pulmonary embolism, and myocardial infarction. The lowest complication rate was among men who underwent surgery 1 - 3 weeks after biopsy while men undergoing RP 4 - 6 weeks after biopsy had the highest complication rate (18.1% and 24.5% respectively; p < 0.001). At one year, men who underwent RP ≤ 3 weeks after biopsy had significantly more incontinence (8.7% vs. 5.8%; p < 0.001) and worse potency rates (31.7% vs. 68.2%; p < 0.001) compared to those undergoing RP > 12 weeks after biopsy. After adjusting for relevant clinicopathologic characteristics, including surgeon, the likelihood of a nerve sparing procedure was highest among those in the groups waiting at least 6 weeks (OR: 1.39; p = 0.01) to 6 months (OR: 1.45, p = 0.02) (Table-2). Patients in the 4 - 6 week group had significantly higher overall complications (OR: 1.33, p = 0.01). Notably, when all surgeons were included in the analysis, the odds of a positive margin (OR: 0.6; p < 0.001) and surgical complication (OR: 0.8; p = 0.03) remained significantly lower among those waiting at least 6 weeks.

**Table 2:** Logistic Regression analysis assessing impact of time from biopsy on peri-operative complications.*

	≤3 weeks	4-6 weeks		7-12 weeks		12 weeks-6 mo		6mo- 1 year	
		OR (95% CI)	P Value	OR (95% CI)	P Value	OR (95% CI)	P Value	OR (95% CI)	P Value
**Nerve sparing (any)**	Ref	1.13 (0.90-1.43)	0.30	1.39 (1.10-1.80)	0.01	1.45 (1.06-1.99)	0.02	0.99 (0.58-1.70)	1.00
**Overall Transfusion**	Ref	1.35 (1.00-1.83)	0.05	1.77 (1.34-2.34)	<0.001	1.77 (1.28-2.44)	0.001	2.04 (1.20-3.40)	0.01
**Overall Complication**	Ref	1.33 (1.07-1.66)	0.01	1.07 (0.86-1.33)	0.57	1.07 (0.83-1.4)	0.58	1.00 (0.66-1.50)	1.00
**Intraop Complication**	Ref	1.66 (0.60-4.91)	0.40	1.19 (0.39-3.64)	0.76	1.63 (0.48-5.58)	0.40	3.14 (0.66-15.04)	0.15
**Post-Op complication**	Ref	1.33 (1.07-1.66)	0.01	1.09 (0.87-1.36)	0.47	1.08 (0.84-1.4)	0.50	1.00 (0.66-1.52)	1.00
**Early Complication**	Ref	1.25 (0.90-1.66)	0.13	1.06 (0.8-1.40)	0.70	1.00 (0.70-1.32)	0.80	1.06 (0.64-1.73)	0.80
**Incontinence**	Ref	1.19 (0.90-1.65)	0.30	1.37 (0.98-1.90)	0.06	1.28 (0.90-1.92)	0.20	1.96 (1.07-3.59)	0.03

* Adjusted for BMI, NCCN risk category, open vs. robotic approach, biopsy Gleason score, clinical stage, PSA, year of surgery, prostate volume, and surgeon

Finally, we compared oncologic outcomes by time from biopsy to surgery by NCCN criteria (Table-3). Median follow-up after RP was 7.1 years (IQR 4.2, 11.7) during which time 1977 (26.9%) men experienced biochemical recurrence. The risk of upgrading increased significantly over time, starting at 7 weeks for low risk men and 4 weeks for intermediate risk men. There was no difference in risk of non - organ confined disease or positive margins. However, among low risk men, the risk of BCR increased significantly for those waiting at least 7 weeks (HR: 1.64; 95% CI: 1.26 - 2.12, p < 0.001) to 6 months (HR: 1.69; 95% CI: 1.25 - 2.28, p = 0.001). For men with high risk disease, waiting more than six months without the use of ADT was associated with an increased risk of BCR (HR: 3.38; 95% CI: 1.0 - 11.44, p = 0.05).

**Table 3:** Multivariate regression of oncologic outcomes among low, intermediate, and high risk men stratified by time from biopsy to surgery, adjusting for clinical T stage, age, PSA, surgeon, and clinical Gleason score, and open vs. robotic approach.

Low Risk								
	Risk of Upgrading		pT3/pT4		Positive Margin		Biochemical Recurrence	
Time in Weeks	OR (95% CI)	P	OR (95% CI)	P	OR (95% CI)	P	HR (95% CI)	P
≤3								
4-6	1.22 (0.84-1.77)	0.3	1.34 (0.80-2.24)	0.27	1.10 (0.77-1.55)	0.61	1.29 (0.97-1.71)	0.07
7-12	1.61 (1.15-2.24)	0.01	1.39 (0.84-2.29)	0.2	1.33 (0.96-1.85)	0.09	1.64 (1.26-2.12)	<0.001
13-26	1.70 (1.19-2.43)	0.003	1.07 (0.59-1.92)	0.83	1.21 (0.83-1.76)	0.32	1.69 (1.25-2.28)	0.001
26-52	2.16 (1.32-3.54)	0.002	1.12 (0.43-2.89)	0.82	1.63 (0.92-2.88)	0.09	1.05 (0.61-1.83)	0.86
Intermediate Risk								
	Risk of Upgrading		pT3/pT4		Positive Margin		Biochemical Recurrence	
Time in Weeks	OR (95% CI)	P	OR (95% CI)	P	OR (95% CI)	P	HR (95% CI)	P
≤3								
4-6	1.52 (1.05-2.21)	0.03	1.15 (0.82-1.61)	0.41	0.86 (0.64-1.16)	0.33	1.07 (0.88-1.31)	0.51
7-12	1.51 (1.04-2.17)	0.03	1.13 (0.81-1.59)	0.47	1.07 (0.79-1.44)	0.66	1.11 (0.91-1.36)	0.31
13-26	1.26 (.81-1.96)	0.31	0.91 (0.6-1.37)	0.64	0.94 (0.64-1.37)	0.73	0.98 (0.75-1.29)	0.9
26-52	2.27 (1.11-4.64)	0.02	0.57 (0.23-1.44)	0.24	1.25 (0.63-2.49)	0.52	0.99 (0.58-1.68)	0.97
High Risk								
	Risk of Upgrading		pT3/pT4		Positive Margin		Biochemical Recurrence	
Time in Weeks	OR (95% CI)	P	OR (95% CI)	P	OR (95% CI)	P	HR (95% CI)	P
≤3								
4-6	1.45 (0.81-2.59)	0.21	0.93 (0.57-1.54)	0.79	1.28 (0.78-2.1)	0.32	1.15 (0.86-1.54)	0.34
7-12	1.02 (0.55-1.87)	0.96	0.94 (0.54-1.63)	0.83	1.04 (0.61-1.79)	0.88	1.35 (0.98-1.87)	0.07
13-26	1.13 (0.49-2.59)	0.78	0.45 (0.2-0.98)	0.04	0.77 (0.35-1.69)	0.52	1.16 (0.71-1.91)	0.55
26-52	4.92 (0.94-25.81)	0.06	0.30 (0.05-1.77)	0.18	2.14 (0.46-10.0)	0.33	3.03 (1.05-8.78)	0.04

## COMMENTS

We examined the association of time from biopsy to RP with complications, functional and oncologic outcomes in a large cohort of men treated with RP in the PSA era. We found that men who underwent early RP were more likely to have clinically higher risk disease and adverse pathologic features at surgery. Perhaps most notably, while RP in the 4 - 6 week time frame was independently associated with increased overall complications, there was no impact on functional outcomes or margin rates.

Following diagnosis by trans - rectal ultrasound - guided biopsy, approximately 50% of men elect to undergo RP for localized prostate cancer (3). Historically, surgery was delayed between 4 and 8 weeks to allow inflammatory adhesions or hematoma to resolve, thus maintaining anatomic relationships between the prostate and the surrounding structures (4, 8). However, other than a lower likelihood of nerve sparing in patients undergoing RP within 6 weeks of biopsy, the impact of time from biopsy to RP on complications and functional outcomes remains controversial (4, 5).

That is, while several studies have reported no differences in complications or positive margin rates (5, 7, 9) others have suggested early RP is associated with more overall complications and a greater risk of blood transfusion (8). Specifically, Eggener et al., in a retrospective series of 2.996 patients analyzed the interval from biopsy to open RP as a dichotomous variable with thresholds of 4 and 6 weeks (5). They found no significant difference in operating time, estimated blood loss, surgical margin status, urinary incontinence or erectile function between the groups on multivariate analysis (5). However, those undergoing RP ≤ 6 weeks after prostate biopsy were significantly less likely to receive a nerve sparing procedure (5).

Using similar time points, Martin et al. examined the effect of time on outcomes in 559 men undergoing RARP between 2004 and 2007 (8). They hypothesized that time from biopsy may have a greater detrimental impact in robotic surgery due to the lack of tactile sense as compared to open prostatectomy (8). In fact, on multivariate analysis they found that an interval between biopsy and surgery of ≤ 6 weeks was associated with a significantly higher rate of complications (P = 0.03) (8). However, they did not find a significant difference in positive margin rates using either the 4 - week (18.5% vs. 22.0%) or 6 - week (22.2% vs. 21.8%) time points (8). Conversely, in a recent retrospective analysis of 1.848 men using 4 weeks as their cutoff for early RP, Park et al. advocated for early minimally invasive RP (≤ 4 weeks after biopsy) after noting decreased operative times amongst this cohort (9).

The exact pathway and mechanism by which the prostate heals following traumatic injury, such a biopsy has not yet been elucidated. The phases of tissue repair in general encompass inflammation, proliferation, and maturation, and the length of each is dependent on numerous factors including tissue type and vascularity. Prior studies have shown that 77% of patients have visible hemorrhage on MRI following biopsy, and this persisted beyond 4 weeks for 49% of men (15). Following hemorrhage, pro - inflammatory cytokines are released which leads to infiltration by circulating inflammatory cells (macrophages, leukocytes) (16). In abdominal surgery, the addition of blood to the peritoneal cavity results in the formation of peritoneal adhesions as clotted blood may form a scaffold on which fibroblasts proliferate, leading to adhesion formation (17, 18). Although controversial, it is hypothesized the Denonvieller's arises from fusion of the two walls of the embryologic peritoneal cul - de - sac (19). We hypothesize that those hematomas which persist beyond 4 weeks are likely larger and potentially more pro - inflammatory as well as more likely to be adherent to Denonvieller's fascia.

Men who undergo early RP for definitive local treatment may represent a distinct population from the broader population diagnosed with prostate cancer. However, even after controlling for the more aggressive clinicopathologic disease in this group as well as surgeon, the increased risk of overall complications persisted in the 4 - 6 week time period. Previously it was hypothesized that trans - rectal needle biopsy causes peri - prostatic inflammation and potentially bleeding and hematoma formation, making identification and dissection of surgical planes more difficult (4, 8). Additionally, tissue reaction from the peri - prostatic nerve block may make neurovascular bundle preservation more difficult and may lead to serious complications such as rectal injury at the time of RP (4, 5). We did not identify any difference in rectal injury between the groups; however, we did find that earlier RP was associated with less nerve sparing.

The impact of delay between diagnosis and surgery on oncologic outcomes is controversial (20–26). Freedland et al. found no difference in BCR rates among low risk men waiting < 3 months versus 3 - 6 months, but reported a higher risk of BCR for those waiting > 6 months (26). Conversely, Vickers et al. found no significant difference in BCR rates between those undergoing RP within 6 months of diagnosis compared to waiting more than 6 months, adjusting for disease severity (7, 25). Boorjian et al. evaluated the impact of delay from diagnosis to treatment among 3.969 men with clinically localized prostate cancer who underwent RP within one year of diagnosis (20). They found waiting up to a year from prostate biopsy to RP did not affect the probability of BCR, even for high risk patients (20).

We found that among low risk men, the risk of upgrading at RP increased with time, consistent with prior studies (21, 27). Surprisingly, low risk men in the 7 - 26 week time period had an increased risk of BCR compared to earlier RP, but those waiting 6 to 12 months did not. This is likely due to men initially considering active surveillance who were subsequently deemed to not be candidates following additional testing such as a prostate MRI or repeat PSA. Finally, in contrast to previous data that has shown that even among high risk men, a delay of up to one year from biopsy to RP does not impact rates of BCR, we found that high risk men waiting more than 6 months had an increased risk of BCR (20, 22).

To our knowledge, we provide here the largest study to date evaluating complications based on time between biopsy and RP. Our large sample size allowed us to group men by time intervals from biopsy as opposed to using a dichotomous time point cut - off. A particular strength is our ability to limit our analysis to the five highest volume surgeons (> 900 cases) while also adjusting for year. This demonstrated that RP in the 4 - 6 weeks after biopsy is associated with a higher overall complication rate while the likelihood of nerve sparing increases with time since biopsy. We also demonstrated that risk of upgrading increases with delay to surgery among low and intermediate risk men. In addition, high risk men waiting more than 6 months until surgery had a higher likelihood of BCR. Our study may have determined a statistically significant difference in complications where multiple prior studies did not due to the larger sample size here, with greater resulting statistical power. Nevertheless, the absolute differences in complication rate were relatively small, and may be of uncertain clinical relevance. Meanwhile, the noted increase in overall complications in the 4 to 6 week time period may be due to post - biopsy peri - prostatic inflammation and hematoma, leading to more difficult dissection of surgical planes among these men. We must acknowledge as well that, given the retrospective nature of our study, the findings may also be due to residual unmeasured confounding or incomplete adjustment for high risk disease.

We recognize that our study was limited by its retrospective design. We do not have complete information about the clinical decision making that prompted earlier versus later intervention following biopsy. Only 28% of the cases were performed robotically as we limited our analysis to the five highest volume surgeons. On multivariable analysis, approach did not impact complications, likely due to surgeon experience. In addition, we do not know whether nerve sparing was intended, only whether it was performed and we do not have information about operative time. Our data set does not collect physician counseling and rationale behind clinical decision making, thus we were unable to adjust for all confounders that may have caused some patients to be operated on within specific time periods. Thus there are likely unmeasured confounders such as patient anxiety, scheduling, or physician perception of higher risk disease. As we are testing multiple hypothesis we must acknowledge the problem of multiple comparisons, specifically that some findings may be statistically significant simply by chance. Thus, we have tried to emphasize in our discussion and take home points only the findings that seem more robust and clinically relevant. Finally, this analysis was limited to the five highest volume prostatectomists at a tertiary referral center and thus the results may not be generalizable to all urologic surgeons. However, overall we believe our findings support the current practice of waiting at least 6 - 8 weeks following biopsy before RP.

## CONCLUSIONS

In conclusion, in this surgical cohort, RP in the 4 - 6 weeks following biopsy was associated with a higher overall complication rate, but no difference in functional outcomes or positive margin rate. While oncologic outcomes for some patient subsets were impacted by the time from biopsy to RP, ultimately there was no difference in local recurrence or systemic progression with a delay of up to one year. If these findings are validated in future studies, this information may be used for surgical planning and to counsel the newly diagnosed prostate cancer patient.

## ABBREVIATIONS

ACM: all - cause mortality
ADT: androgen deprivation therapy
BCR: biochemical recurrence
CSM: cancer - specific mortality
IQR: Interquartile Range
PCa: prostate cancer
RP: radical prostatectomy
PSA: prostate specific antigen
PFS: systemic progression - free survival
TRUS: Trans rectal ultrasound
